# Impact of Neuromuscular Fatigue on Dynamic Knee Valgus in Female Basketball Players

**DOI:** 10.3390/life15050816

**Published:** 2025-05-20

**Authors:** Beatriz B. Gomes, Ricardo Cardoso, Rui A. Fernandes, Rui A. Ferreira

**Affiliations:** 1 University of Coimbra, CIPER (Interdisciplinary Center for the Study of Human Performance), FCDEFUC (Faculty of Sports Science and Physical Education), 3040-248 Coimbra, Portugal; rui.fernandes@uc.pt (R.A.F.); ruiarantesferreira@gmail.com (R.A.F.); 2 University of Coimbra, CEMMPRE (Centre for Mechanical Engineering, Materials and Processes), ARISE (Advanced Production & Intelligent Systems–Associated Laboratory), 3030-788 Coimbra, Portugal; 3 University of Porto, CIFI2D (Centre of Research, Education, Innovation and Intervention in Sport), LABIOMEP (Porto Biomechanics Laboratory), Faculty of Sport, 4200-450 Porto, Portugal; ricardocardoso.coach@gmail.com

**Keywords:** biomechanics, single-leg drop jump, change of direction, frontal plane projection angle (FPPA)

## Abstract

Dynamic knee valgus is a biomechanical condition often linked to an increased risk of knee injuries, particularly in female athletes, due to greater hip adduction, internal rotation, and knee abduction during dynamic movements. This study aimed to assess the impact of neuromuscular fatigue on dynamic knee valgus in female basketball players during single-leg drop jumps (DJ-SL) and change of direction (COD) tests at 45° and 90°. Thirty-three athletes, divided into national and regional performance groups, performed these movements before and after a fatigue protocol. Fatigue was induced through a series of anaerobic exercises, and frontal plane projection angle (FPPA) was used to measure knee valgus. The results showed that dynamic knee valgus increased with the angle of directional change (from 24.77° ± 8.25 at 45° to 34.55° ± 10.40 at 95° pre-fatigue, and from 26.59° ± 12.30 at 45° to 35.87° ± 10.37 post-fatigue), but was not significantly affected by neuromuscular fatigue. The national group demonstrated lower valgus angles compared to the regional group, indicating potential performance differences based on competitive level. These findings suggest that while neuromuscular fatigue does not notably impact knee valgus, the higher valgus angles during directional changes warrant attention in injury prevention programs for female basketball players. Further research is needed to explore other factors influencing knee mechanics and injury risk.

## 1. Introduction

Knee injuries are common in high-intensity sports such as basketball, which involve frequent jumping, directional changes, and landings. Female athletes are four to six times more likely to suffer non-contact anterior cruciate ligament (ACL) injuries than their male counterparts [1], mainly due to anatomical, hormonal, and neuromuscular differences [1,2,3,4,5,6,7]. These differences are also associated with higher levels of dynamic knee valgus (DKV) in female athletes. DKV, defined as the medial collapse of the knee during weight-bearing activities, is one of the main biomechanical risk factors for knee injuries [8]. It is strongly associated with ACL injuries and other knee pathologies, highlighting its importance in injury prevention and screening strategies [3,9,10,11].

Recent research has focused on understanding how different conditions, such as fatigue, affect knee mechanics and increase the risk of injury [12]. Neuromuscular fatigue has been shown to impair neuromuscular control and alter movement patterns, potentially exacerbating dynamic knee valgus during athletic activities [10,13]. However, the impact of lower limb neuromuscular fatigue on DKV during specific basketball movements, such as single-leg jumps (DJ-SL) and change of direction (COD) tests, remains less clear. While some studies suggest that lower limb neuromuscular fatigue increases knee valgus [5,14], others indicate that athletes may adapt their movements to protect against excessive knee strain [15]. Despite these findings, there is still a lack of research focusing specifically on COD movements in young female basketball players. In particular, the fast, multidirectional movements inherent to basketball, such as quick pivots and sudden direction changes, make this sport particularly relevant for examining DKV. Few studies have examined how different angles of directional change (e.g., 45° vs. 90°) influence fatigue-related alterations in knee mechanics, or how responses may vary between athletes of different competitive levels.

The specific hypothesis of this study is that lower limb neuromuscular fatigue exacerbates dynamic knee valgus during both DJ-SL and COD movements at 45° and 90°, with larger increases in DKV expected for athletes at the regional level compared with those at the national level. Given the high incidence of lower extremity injuries in basketball, exceeding 65% [16], this study addresses a relevant gap by investigating how lower limb neuromuscular fatigue influences dynamic knee valgus during DJ-SL and COD tasks at 45° and 90°, comparing national- and regional-level female basketball players. Knee injuries are the second most common type of injury among players under 14 years old [17], with non-contact ACL injuries being particularly prevalent [7]. Additionally, lower limb neuromuscular fatigue has been shown to exacerbate joint stress, increasing the risk of injury [3].

Understanding how fatigue affects these movements is crucial for informing injury prevention programs designed to reduce knee injury risk in this population. The results of this study can provide insight into whether fatigue-specific training interventions may be beneficial in improving knee stability and preventing injuries in female athletes.

## 2. Materials and Methods

### 2.1. Participants

This study involved 33 female basketball players (aged 14–16 years) with no known lower limb injuries at the time of the study, from four different teams, divided into two performance groups: national (n = 17, height 168.00 ± 5.44 cm and weight 62.59 ± 9.95 kg) and regional (n = 16, height 164.56 ± 8.11 cm and weight 55.13 ± 8.02 kg). These two groups differed in performance level: the National group consisted of players from clubs that participated in the highest-level competition of this category, while the Regional group was made up of teams that were unable to access that level. Participants were selected based on their involvement in the U16 competition. Participants were selected using a convenience sampling method, based on availability from local teams. Due to logistical constraints, no formal power calculation was performed for the sample size. All athletes and their legal guardians provided written informed consent before participation, following the Declaration of Helsinki. Ethical approval for this study was obtained from the Local Scientific Committee.

### 2.2. Study Design

This study was designed as a pre–post test experiment to assess the effect of lower limb neuromuscular fatigue on DKV in female basketball players. The study involved biomechanical testing of athletes during DJ-SLs and COD tests (at 45° and 90° angles) before and after a fatigue-inducing protocol. The frontal plane projection angle (FPPA) was measured as an indicator of DKV under neuromuscular non-fatigued and fatigued conditions. This study was conducted over two sessions for each team, with all tests performed during regular training hours at the athletes’ local sports facilities.

### 2.3. Procedures

**Figure 1:**
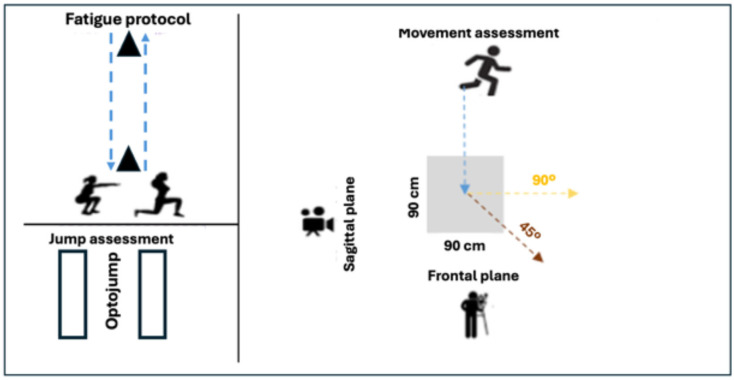
Testing layout representing the fatigue protocol, jump assessment, movement assessment and camera positioning.

**Figure 2:**
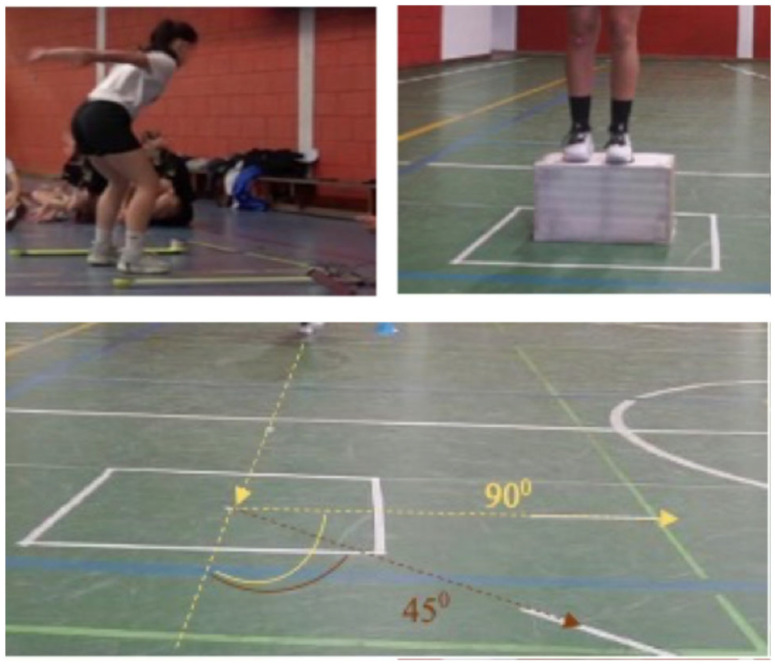
Optojump (CMJ free arms), one leg drop jump (DJ-SL) 30 cm, and changes of direction (90° and 45°) (upper left, right and bottom panels, respectively).

All athletes performed a standardized 15 min warm-up before the tests, which included dynamic stretching and sport-specific movements (e.g., jumps and direction changes). Following the warm-up, participants completed a pre-test of three trials of countermovement jumps (CMJ) to assess baseline power output using the Optojump system (Microgate, Bolzano, Italy) (Figure 1 and Figure 2).

#### 2.3.1. Single-Leg Drop Jumps (DJ-SL)

Athletes performed single-leg drop jumps from a 30 cm box using their dominant leg (self-reported) before the lower limb neuromuscular fatigue protocol. The drop jump involved stepping off the box and immediately jumping upward upon landing. DKV was assessed by measuring the maximum FPPA during the landing phase, captured by video analysis, as described below (Figure 1, upper left panel).

#### 2.3.2. Change of Direction (COD) Test

The COD test was performed by having athletes run 4 m toward a designated 90 × 90 cm square, where they had to execute CODs at angles of 45° and 90°. Athletes performed these direction changes at maximum speed, ensuring foot placement within the designated area. Each participant completed two trials for both COD tests (45 and 90°) before (BF) and after (AF) the fatigue protocol. The FPPA was measured during the execution of these movements using synchronized video analysis to assess DKV. The FPPA was recorded at the moment of peak knee flexion during the COD, providing insights into how fatigue and movement intensity affect knee alignment (Figure 1, bottom panel).

#### 2.3.3. Frontal Plane Projection Angle (FPPA) and Video Analysis

The FPPA was used in this study to assess dynamic knee alignment during athlete movements, specifically during the DJ-SL and COD tasks. FPPA was measured before and after the muscle fatigue protocol to evaluate the effects of fatigue on knee kinematics.

**Figure 3:**
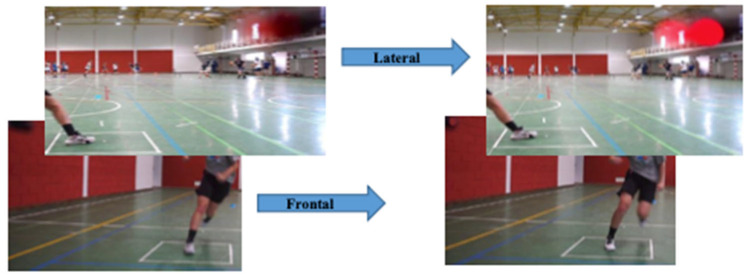
Synchronization of the two cameras using the LED light (left frame before switching on the light and right frame after switching on the light), representing the two planes (side and front).

Two high-resolution, synchronized digital video cameras (SONY ILCE-5100L and CYBER-SHOT RX100 M5 A, Tokyo, Japan) were positioned 3 m from the test area. One camera was placed in front of the athlete to capture the frontal plane, and the second was positioned laterally to capture the sagittal plane. Both cameras recorded at a frame rate of 60 Hz to allow for accurate frame-by-frame motion analysis (Figure 3).

**Figure 4:**
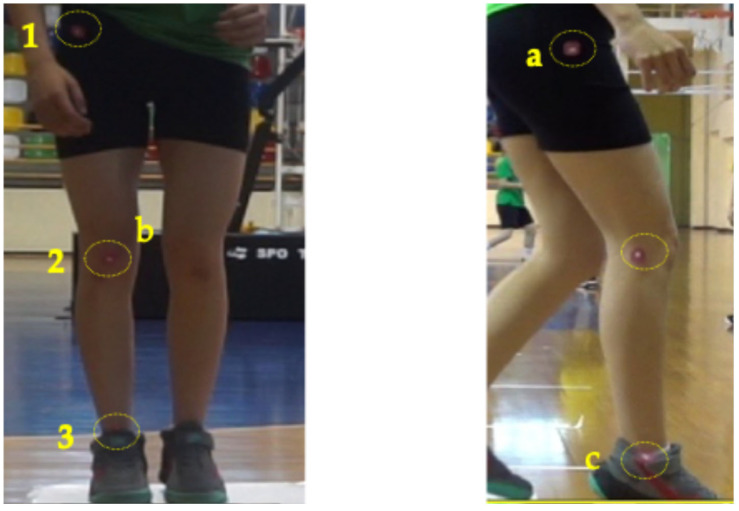
Anatomical landmark positions for FPPA analysis: (1) ASIS, (2) center of the patella and (3) midpoint between the medial and lateral malleolus (left panel); B for sagittal plane knee angle analysis: (a) greater trochanter of the femur, (b) femorotibial joint space and (c) tibial lateral malleolus (right panel).

For FPPA analysis, reflective markers were placed on anatomical landmarks, namely the anterior superior iliac spine (ASIS), the center of the patella (knee), and the midpoint between the medial and lateral malleolus (ankle) (Figure 4). For sagittal plane knee angle analysis, markers were placed on the greater trochanter of the femur (hip joint), femorotibial joint space (knee), and tibial lateral malleolus (ankle) (Figure 4).

FPPA was calculated as the angle formed by two segments: one from the ASIS to the center of the patella (representing the thigh segment) and the other from the center of the patella to the midpoint between the medial and lateral malleolus (representing the lower leg segment). The angle was measured at the point of maximum knee flexion during the landing phase of the DJ-SL and the cutting phase of the COD tasks. The sagittal plane recording was used to identify the frame corresponding to peak knee flexion, and this frame was then used from the frontal camera to assess the FPPA.

Each movement trial was analyzed independently, and three successful trials were recorded for each condition (pre- and post-fatigue). The average of these two trials was used for statistical analysis. All angle measurements were performed using Tracker software (version 5.1.5), a validated motion analysis tool commonly used for 2D biomechanical assessments [18]. The relevance and feasibility of using 2D motion capture systems for joint angle measurement have been demonstrated in recent studies, particularly in the context of DKV research [19,20]. To ensure reliability, 15% of the videos were randomly selected and re-analyzed by the same rater two weeks later to calculate intra-rater reliability, which showed excellent agreement (intraclass correlation coefficient, ICC > 0.91).

#### 2.3.4. Fatigue Protocol

The fatigue protocol consisted of two sets of 10 squat jumps, 10 lunge jumps, and a 10 m sprint. After each set, CMJ performance was reassessed using the Optojump system (Microgate, Bolzano, Italy, software version V1.12.21.0) to verify a reduction of at least 20% in vertical jump height compared to the baseline measurement taken at the beginning of the testing protocol. This 20% threshold was adopted based on previous research indicating that such a decrease reflects a meaningful level of neuromuscular fatigue affecting performance capacity [21]. If the fatigue criterion was not met, additional sets were performed.

### 2.4. Statistical Analysis

All statistical analyses were performed using SPSS (version 27.0). Descriptive statistics (means and standard deviations) were calculated for all variables. The Shapiro–Wilk test was used to assess the normality of the data distribution for all dependent variables. Paired sample *t*-tests were used to compare pre- and post-fatigue FPPA values. Independent samples *t*-tests were conducted to compare the National and Regional groups. The effect size (Cohen’s d) was calculated for the comparisons. Pearson correlation coefficients were used to examine relationships between variables such as FPPA and performance measures. Statistical significance was set at *p* < 0.05.

## 3. Results

Table 1 presents the mean and standard deviation values of the DKV angles measured through the FPPA before and after the fatigue induction protocol. Following the fatigue protocol, the DKV during the COD test increased by 1.82° (approximately 7%) for the 45° condition and by 1.32° (approximately 4%) for the 90° condition. However, these increases were not statistically significant, with *p* = 0.318 for 45° and *p* = 0.416 for 90°, as confirmed by small effect sizes (ES, d < 0.2), indicating minimal practical relevance.

**Figure 5:**
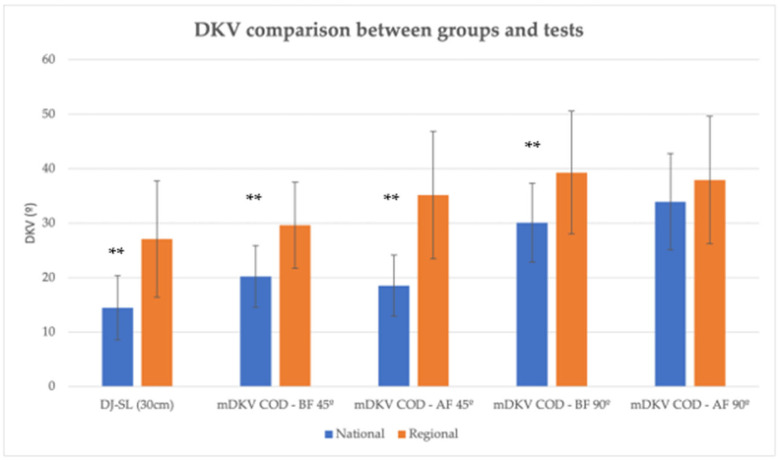
Dynamic knee valgus (means and SDs) comparison between National and Regional groups for single leg drop jump (DJ-SL) and change of direction (COD) for 45 and 90 degrees, before (BF) and after (AF) fatigue induction protocol. ** statistically significant differences (*p* < 0.01).

Table 2 presents a comparison between the National and Regional groups, highlighting differences between the groups in the selected variables. Differences were observed in the DKV measured in the DJ-SL test (*p* = 0.000), in the 45° COD before and after fatigue (*p* = 0.000), in the 90° COD before the fatigue protocol (*p* = 0.008), in the maximum power achieved during the RAST test (*p* = 0.003), and in the fatigue index during the RAST test (*p* = 0.000). Although no differences were found in DKV after the fatigue protocol or in the 90° COD, it was observed that valgus values were lower in the National group than in the Regional group (Figure 5). In the RAST test, maximum power was significantly higher in the National group, with a significant difference and a large effect size (d > 0.8). Although the minimum power did not present significant differences, it was also higher in the National group. Additionally, the National group demonstrated a higher fatigue index in this test, while a lower fatigue index was observed in the Regional group (*p* = 0.000).

**Figure 6:**
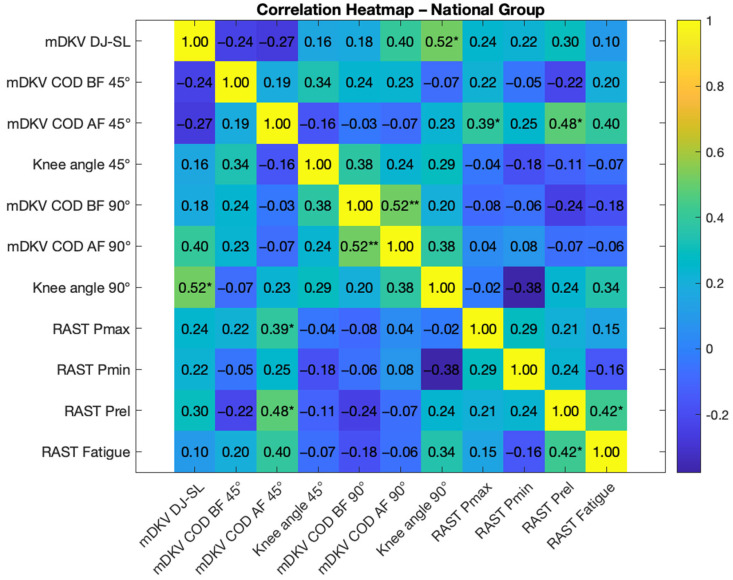
Pearson correlation heatmap for the National group results on the different tests and variables analyzed, statistically significant differences * *p* < 0.05, ** *p* < 0.01. Legend: mDKV DJ-SL—maximum dynamic knee valgus during the single leg drop jump (30 cm); mDKV COD—BF 45°—maximum dynamic knee valgus during the execution of the 45° COD before fatigue induction protocol; mDKV COD—AF 45°—maximum dynamic knee valgus during the execution of the 45° COD after fatigue induction protocol; knee angle 45°—relative knee angle at DKV during the execution of the 45° COD; mDKV COD—BF 90°—maximum dynamic knee valgus during the execution of the 90° COD before fatigue induction protocol; mDKV COD—AF 90°—maximum dynamic knee valgus during the execution of the 90° COD after fatigue induction protocol; knee angle 90°—relative knee angle at maximum DKV during the execution of the 90° COD; RAST Pmax—maximum power in the RAST test; RAST Pmin—minimum power in the RAST test; RAST fatigue—fatigue index during the RAST test; CMJ FREE ARMS PF—percentage reduction in flight time during the countermovement jump test with free arm movement after the fatigue induction protocol.

**Figure 7:**
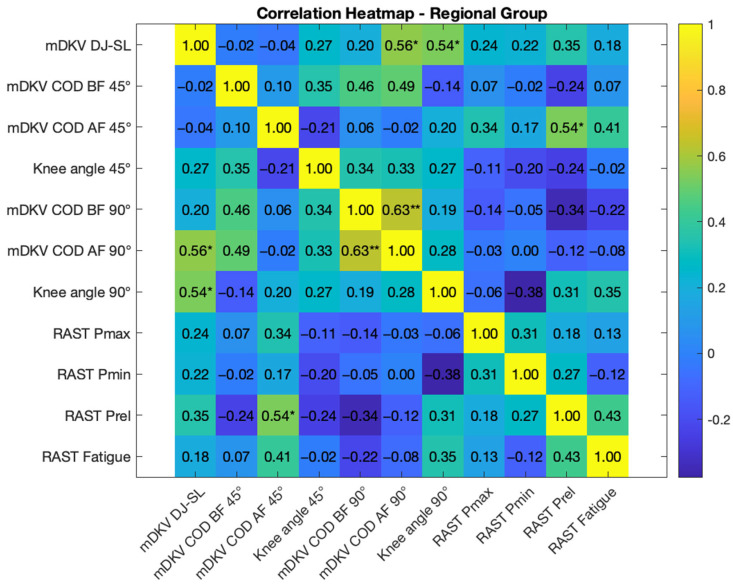
Pearson correlation heatmap for the Regional group results on the different tests and variables analyzed, statistically significant differences * *p* < 0.05, ** *p* < 0.01. Legend: mDKV DJ-SL—maximum dynamic knee valgus during the single leg drop jump (30 cm); mDKV COD—BF 45°—maximum dynamic knee valgus during the execution of the 45° COD before fatigue induction protocol; mDKV COD—AF 45°—maximum dynamic knee valgus during the execution of the 45° COD after fatigue induction protocol; knee angle 45°—relative knee angle at DKV during the execution of the 45° COD; mDKV COD—BF 90°—maximum dynamic knee valgus during the execution of the 90° COD before fatigue induction protocol; mDKV COD—AF 90°—maximum dynamic knee valgus during the execution of the 90° COD after fatigue induction protocol; knee angle 90°—relative knee angle at maximum DKV during the execution of the 90° COD; RAST Pmax—maximum power in the RAST test; RAST Pmin—minimum power in the RAST test; RAST fatigue—fatigue index during the RAST test; CMJ FREE ARMS PF—percentage reduction in flight time during the countermovement jump test with free arm movement after the fatigue induction protocol.

Figure 6 and Figure 7 present the Pearson correlation analysis between the studied variables for the National and Regional level groups, respectively.

In the National group, the maximum dynamic knee valgus during the single leg drop jump results correlated with the knee angle during the 45° COD (r = 0.601, *p* < 0.05), while in the Regional group it was associated with dynamic knee valgus during the 90° COD after the fatigue induction protocol (r = 0.561, *p* < 0.05), as well as with the relative knee angle in the same execution (r = 0.537, *p* < 0.05). Dynamic knee valgus during the 45° COD showed a highly significant relationship with valgus during the same movement after the fatigue protocol in the National group (r = 0.715, *p* < 0.01). In the Regional group, dynamic knee valgus during the 45° COD was related to relative power in the RAST test (r = 0.535). Dynamic knee valgus during the 90° COD also showed a positive relationship (r = 0.508, *p* < 0.05) with valgus in the same test after the fatigue induction protocol. This latter valgus was also related to maximum power (r = 0.506, *p* < 0.05) and the fatigue index (r = 0.503, *p* < 0.05), both in the RAST test, within the National group. In the Regional group, dynamic knee valgus during the 90° COD was correlated (r = 0.631, *p* < 0.01) with valgus in the same test after the fatigue protocol, as well as with dynamic knee valgus in the DJ-SL test, as mentioned earlier.

## 4. Discussion

The current study evaluated dynamic knee valgus (DKV) during DJ-SL and COD movements in female basketball athletes, examining fatigue’s impact. Significant differences in DKV were observed between the National (14.46 ± 5.88°) and Regional (27.07 ± 10.67°) groups during the DJ-SL test. In the National group, DKV increased from 14.46 ± 5.88° in the DJ-SL to 20.21 ± 5.67° during the 45° COD, and further to 30.07 ± 7.26° during the 90° COD. In contrast, the Regional group showed an increase from 27.07 ± 10.67° in the DJ-SL to 29.61 ± 7.90° at 45° COD, and 39.30 ± 11.30° at 90° COD. These increases in DKV with greater changes in direction align with findings by Sinsurin et al. [22], who observed that as the angle of COD increases, the DKV also tends to increase, particularly in sports movements requiring high agility and directional shifts.

Regarding dynamic knee valgus, an increase was observed following the neuromuscular fatigue induction protocol, both during the 45° and the 90° change of direction, which is consistent with previous findings. However, no statistically significant differences were observed under fatigue in the current study [7,8]. When analyzing the data based on the athletes’ competitive level, notable differences were found, such as changes in DKV due to fatigue. In the National group, DKV during the 45° change of direction was reduced after the neuromuscular fatigue induction protocol. In contrast, a reduction was observed in the Regional group during the 90° change of direction, from before to after the fatigue protocol. Although such reductions in dynamic knee valgus following fatigue protocols are not the norm in most studies, they are not unprecedented, as they have been previously described. This may suggest that fatigue can trigger protective neuromuscular adaptations to minimize joint loading, as it has also been reported that the knee angle at the moment of contact tends to decrease under fatigue [6].

It is also important to highlight that DKV values during the 90° COD in the Regional group approached the upper threshold of reported ranges in previous studies, with 39° of DKV [2]. Nonetheless, it should be noted that 2D video analysis methods, such as the FPPA used in this study, may overestimate valgus angles when compared to 3D motion capture systems [23,24]. This overestimation is particularly relevant in athletes with poorer motor control and greater transverse plane deviations, which can project the knee more medially in the frontal plane [25]. Therefore, while the recorded values are high, they should be interpreted considering the methodological limitations of a 2D assessment.

In the current study, dynamic knee valgus during the DJ-SL showed a positive correlation with the knee angle during the 45° change of direction under fatigue (r = 0.601, *p* < 0.05) for the National group, and with the knee angle during the 90° change of direction under fatigue (r = 0.561, *p* < 0.05) for the Regional group. Interestingly, this correlation occurs in movements where dynamic knee valgus decreased after the fatigue protocol in the respective groups, possibly influenced by inter-joint coordination or even micro-adjustments, although not directly measured in this study [5]. This relationship suggests that a reduction in dynamic knee valgus occurs in athletes who also tend to have lower relative knee angle values [13]. Such reductions may reflect compensatory strategies to mitigate fatigue-related joint instability, likely resulting in reduced performance.

In addition, in the National group, there was a highly significant relationship (r = 0.715, *p* < 0.01) between dynamic knee valgus before and after the fatigue protocol during the 45° change of direction, where valgus decreased. The Regional group also showed a correlation during the 90° change of direction (r = 0.631, *p* < 0.01), where the valgus also decreased. Regarding anaerobic muscular power assessment via the RAST, the measured values indicate that the National group can generate greater power than the Regional group, both in absolute and relative terms. However, the only statistically significant result was the difference in the fatigue index, which was higher in the National group compared to the Regional group. The athletes’ training backgrounds may influence these findings, as higher power output may result in greater neuromuscular fatigue and metabolic stress, which in turn can impair proprioception, reduce neuromuscular control, and lead to less knee extension and greater valgus angles during jumps [10,11,12].

Nonetheless, a higher training status might enhance motor control strategies that help compensate for fatigue-related impairments, potentially reducing injury risk despite higher fatigue levels [13]. The importance of targeted training interventions, such as gluteal strengthening, core stability, and ankle dorsiflexion range of motion, should be emphasized to correct faulty movement patterns and reduce injury risk as part of a prevention program [14].

Some limitations of the current study include: (i) due to a stop on the championship, some players did not maintain their training regime during this phase; (ii) during the neuromuscular fatigue protocol, the movement velocity was not controlled, even though it was specifically requested to be performed at maximum velocity; (iii) the DKV values obtained via FPPA 2D analysis may be overestimated compared to those from 3D motion capture systems, particularly in individuals with altered transverse plane mechanics or postural compensations; and (iv) more familiarization sessions should have been conducted to reduce variability due to learning effects.

Future research should incorporate force plates to measure contact time, ensure tighter control over execution speed, and use 3D motion capture systems for more comprehensive biomechanical analysis. Additionally, future discussions should explore the underlying physiological and biomechanical mechanisms to explain group differences and unexpected trends in fatigue responses.

## 5. Conclusions

The results indicate that the DJ-SL movement generally leads to less dynamic knee valgus than the change of direction (COD) test. In contrast, the COD test tends to increase dynamic knee valgus due to the larger angle involved. Furthermore, the findings suggest that fatigue can alter dynamic knee valgus, especially during the COD test.

Notably, during the 45° directional change, the National group exhibited decreased valgus, whereas the Regional group showed an increase. Conversely, during the 90° directional change, the National group demonstrated an increase in dynamic knee valgus, while the Regional group displayed a decrease.

This study was unable to objectively determine the factors underlying these divergent results. Additionally, the knee dynamic valgus values recorded in the DJ-SL were lower than those measured during the COD test, although no significant correlation was found between these two variables. The assessment of physical condition through the RAST did not reveal any relationship with dynamic knee valgus values (DJ-SL and COD), despite observed differences in maximum power and fatigue index between the National and Regional groups. This suggests that the RAST should not be regarded as the sole predictor of a predisposition to dynamic knee valgus.

The challenges in correlating the various factors underscore the variability in athletes’ responses to similar movements, highlighting the necessity of considering additional influencing factors. Although the dynamic knee valgus values obtained during the DJ-SL were lower, those recorded during the directional changes approached the maximum values reported in other studies.

## Figures and Tables

**Table 1:** Descriptive analysis (means and standard deviations) and comparisons (dependent samples *t*-test and respective effect size) before and after the fatigue induction protocol for the maximum DKV results in the COD conditions 45° and 90°.

Variable	Before Fatigue N = 33	After Fatigue N = 33	*t*-Test (*p*)	Effect Size
mDKV—COD 45° (°)	24.77 ±8.25	26.59 ± 12.30	−0.318 (0.552)	0.176
mDKV—COD 90° (°)	34.55 ± 10.40	35.87 ± 10.37	−0.416 (0.602)	0.143

mDKV—COD 45°—maximum dynamic knee valgus during the execution of the 45° COD test; mDKV—COD 45°—maximum dynamic knee valgus during the execution of the 90° COD test.

**Table 2:** Descriptive analysis (means and standard deviations) and comparisons (independent samples *t*-test and respective effect size) were conducted based on the group (National and Regional) for performance on the DJ-SL test, COD, RAST, and response to the fatigue induction protocol assessed using the CMJ free arms.

	Variable	National N = 17	Regional N = 16	*p*	ES
	mDKV DJ-SL (30 cm) (°)	14.46 ± 5.88	27.07 ± 10.67	0.000 **	1.476
**Change of Direction (COD)**	mDKV COD—BF 45° (°)	20.21 ± 5.67	29.61 ± 7.90	0.000 **	1.373
	mDKV COD—AF 45° (°)	18.55 ± 5.61	35.14 ± 11.73	0.000 **	1.823
	Knee angle 45 (°)	133.72 ± 28.87	128.57 ± 14.85	0.528	0.222
	mDKV COD—BF 90 (°)	30.07 ± 7.26	39.30 ± 11.30	0.008 **	0.979
	mDKV COD—AF 90 (°)	33.93 ± 8.85	37.93 ± 11.72	0.275	0.387
	Knee angle 90° (°)	136.91 ± 12.71	128.73 ± 12.81	0.075	0.641
	RAST Pmax (W)	409.29 ± 86.76	326.53 ± 52.75	0.003 **	1.144
	RAST Pmin (W)	235.23 ± 60.17	224.38 ± 38.88	0.560	0.205
	RAST Prel (W/kg)	6.62 ± 1.38	5.99 ± 1.06	0.156	0.507
	RAST Fatigue (W/s)	4.51 ± 1.60	2.68 ± 0.91	0.000 **	1.396
	CMJ FREE ARMS PF (%)	24.51 ± 4.74	22.34 ± 2.05	0.101	0.574

Legend: mDKV DJ-SL—maximum dynamic knee valgus during the single leg drop jump (30 cm); mDKV COD—BF 45°—maximum dynamic knee valgus during the execution of the 45° COD before fatigue induction protocol; mDKV COD—AF 45°—maximum dynamic knee valgus during the execution of the 45° COD after fatigue induction protocol; knee angle 45°—relative knee angle at DKV during the execution of the 45° COD; mDKV COD—BF 90°—maximum dynamic knee valgus during the execution of the 90° COD before fatigue induction protocol; mDKV COD—AF 90°—maximum dynamic knee valgus during the execution of the 90° COD after fatigue induction protocol; knee angle 90°—relative knee angle at maximum DKV during the execution of the 90° COD; RAST Pmax—maximum power in the RAST test; RAST Pmin—minimum power in the RAST test; RAST fatigue—fatigue index during the RAST test; CMJ FREE ARMS PF—percentage reduction in flight time during the countermovement jump test with free arm movement after the fatigue induction protocol; ** statistically significant differences (*p* < 0.01); ES—effect size. The grey area represents the analysis of change of direction variables.

## Data Availability

Data are contained within the article.
